# The Role of Inflammation in the Pathogenesis of Macular Edema Secondary to Retinal Vascular Diseases

**DOI:** 10.1155/2014/432685

**Published:** 2014-07-22

**Authors:** Francisco J. Ascaso, Valentín Huerva, Andrzej Grzybowski

**Affiliations:** ^1^Department of Ophthalmology, “Lozano Blesa” University Clinic Hospital, San Juan Bosco 15, 50009 Zaragoza, Spain; ^2^Aragon Health Sciences Institute, Zaragoza, Spain; ^3^Department of Ophthalmology, University Hospital Arnau de Vilanova, Lleida, Spain; ^4^IRB-Lleida, Lleida, Spain; ^5^Department of Ophthalmology, Poznań City Hospital, Poznań, Poland; ^6^University of Warmia and Mazury, Olsztyn, Poland

## Abstract

Macular edema (ME) is a nonspecific sign of numerous retinal vascular diseases. This paper is an updated overview about the role of inflammatory processes in the genesis of both diabetic macular edema (DME) and ME secondary to retinal vein occlusion (RVO). We focus on the inflammatory mediators implicated, the effect of the different intravitreal therapies, the recruitment of leukocytes mediated by adhesion molecules, and the role of retinal Müller glial (RMG) cells.

## 1. Macular Edema: A Nonspecific Indication of Numerous Retinal Vascular Disorders

Macular edema (ME) is defined as an accumulation of either extracellular (mainly in the outer plexiform and the inner nuclear layers) or intracellular fluid (swelling of retinal Müller glial (RMG) cells) in the central part of the retina. Indeed, at times, a combination of these types of fluid accumulation occurs [1]. ME is a nonspecific sign of numerous retinal vascular diseases, such as diabetic retinopathy (DR) and retinal vein occlusions (RVO) [2, 3]. In these disorders, inflammatory processes have been considered to be critical [4–6], and breakdown of the blood retinal barrier (BRB) coupled to the subsequent increase in vascular permeability often causes ME and concomitant visual acuity impairment, secondary to an increased flux in the retinal capillary endothelial cells [7, 8]. Thus, the pathogenesis of diabetic macular edema (DME) includes several interrelated factors such as chronic hyperglycemia, hypoxia, accumulation of free radicals, activation of vascular endothelial growth factor (VEGF), alterations in endothelial intercellular junctions, pericyte loss, retinal vessel leukostasis, disruption of the BRB, and an increase in vascular permeability [9, 10]. Although the pathogenesis of ME when associated with RVO (RVO-ME) is not fully understood, increased rigidity of a crossing artery as a result of an atherosclerotic process has been suggested to cause compression of the underlying vein, provoking turbulent blood flow, endothelial damage, and thrombus formation [11]. Likewise, a common vitreous adhesion at the obstruction site has also been reported, suggesting a possible role of vitreovascular traction in the etiology of some cases of BRVO [12, 13].

Atherosclerosis is a chronic low-grade inflammatory disorder and inflammation within the vascular wall contributes to the development of ME [14–16]. Due to BRB breakdown secondary to damage at the tight junctions of endothelial cells, fluid diffusion from the occluded veins into the tissue can lead to ME [17]. In addition, through such mechanisms, inflammatory responses and vascular dysfunction can all interact to cause retinal ischemia, which induces the expression of VEGF [18]. DME and BRVO-ME may differ in terms of pathogenesis because the cytokine concentrations in the aqueous humor are quite different, suggesting that the inflammatory reaction may be more activated in DME than in BRVO-ME, and ischemic insult may play a central role in the development of BRVO-ME [19].

## 2. The Role of Inflammatory Mediators in the Pathogenesis of Macular Edema

Since Vinores et al. [20] first described the role of VEGF in both ischemic and inflammatory ocular pathologies, it is well known that certain inflammatory mediators are present at the sites of ME, such as the aforementioned VEGF, together with cytokines, chemokines, angiotensin II, prostaglandins, matrix metalloproteinases, interleukins, selectins, vascular cell adhesion molecule-1 (VCAM-1), intercellular adhesion molecule-1 (ICAM-1), and inflammatory cells (macrophages and neutrophils), all of which participate in a complex chain of events that has yet to be fully defined [21, 22]. The vitreous levels of these inflammatory factors appear to be related to the pathological processes [23], although it remains to be seen what blood components are extravasated, how and where they flow into the retinal tissue, and from which vessels they are absorbed [24].

It is important to define which inflammatory mediators are enhanced or dampened in the clinical situation. Indeed, it is known that the concentration of several cytokines in the vitreous cavity increases in eyes with BRVO-ME [25–27], including VEGF and interleukin-6 (IL-6), and that such increases are related to the severity and prognosis of ME [28]. Likewise, increased vitreous fluid levels of interleukin-6 (IL-6), monocyte chemotactic protein-1 (MCP-1), pigment epithelium-derived factor (PEDF), and particularly VEGF and ICAM-1 were related to retinal vascular permeability and the severity of DME [29].

However, whereas the aqueous humour is easily accessible and can be examined even in an outpatient setting, it is not possible to evaluate the vitreal levels of these cytokines in a routine examination [30]. When the vitreous levels of VEGF and interleukin-6 (IL-6) have been measured in patients with DME or with ME due to BRVO and CRVO, the vitreal VEGF concentration proved to be very similar in each group [31]. However, the level of IL-6 in the vitreous cavity was significantly higher in DME patients than in those with BRVO or CRVO. Noma et al. investigated whether VEGF or IL-6 contributes to the pathogenesis of ME in eyes with BRVO [26] and CRVO [32]. They found that the vitreous fluid level of VEGF was significantly higher in the patients with BRVO and CRVO than in controls. The vitreous fluid level of IL-6 was also significantly higher in the patients with both types of RVO than in the control subjects. In the BRVO and CRVO patients, there was a significant correlation between the vitreous levels of VEGF and IL-6. Vitreous fluid levels of both VEGF and IL-6 were significantly higher in patients with BRVO/CRVO patients with ischemia than in those without ischemia. In addition, the vitreous levels of both factors were significantly correlated with the severity of macular edema in the BRVO/CRVO patients. Nevertheless, further studies will be needed to fully understand the relationship of certain inflammatory mediators to DME and ME secondary to BRVO or CRVO.

## 3. Recruitment of Leukocytes Mediated by Adhesion Molecules

Chemokines are multifunctional mediators that can recruit leucocytes to sites of inflammation, promoting further inflammation [33, 34]. The vitreous levels of some chemokines, including MCP-1 and MIP-1a and MIP-1b, have been reported to be affected by different retinal diseases, including DME and RVO [35–37]. Mononuclear cell chemoattractants, such as MCP-1, IL-1, IL-6, IL-8, IL-12, and TNF-*α*, are also known to be expressed in ischemic areas, and these factors may induce the recruitment of leukocytes and their adhesion to the target tissue [38]. Thus, it may not be surprising that MIP-1b is expressed in eyes with DME and ME-RVO given that these disorders lead to retinal ischemia and inflammation [19, 36, 37, 39].

Leukocytes also play a role in increasing vascular permeability, along with VEGF. When they accumulate in the perivascular space, monocytes and lymphocytes initiate this process through leucocyte endothelial interactions [40]. These interactions are mediated by adhesion molecules (selectins, immunoglobulins, integrins, etc.) expressed by the vascular endothelium [41], which contribute to the disruption of tight junctions and the breakdown of the BRB [42, 43]. BRB breakdown may be initiated by different mechanisms, including leucocyte-mediated (recruitment and adhesion) endothelial injury, changes in endothelial cells, activation of protein kinase C, and the induction of fenestrations and vesiculovacuolar organelles [1].

## 4. The Role of Retinal Müller Glial (RMG) Cells

It is well known that ME develops due to vascular leakage and/or through cytotoxic events (e.g., glial cell swelling) [44, 45]. Although their importance in retinal vascular diseases is not fully known, RMG cells play a crucial role in regulating the volume of the extracellular space and water and ion homeostasis and in preserving the inner BRB [46].

Excess water is absorbed by retinal pigment epithelium (RPE) and RMG cells. RPE cells carry out the subretinal fluid, whereas RMG cells dehydrate the inner retinal tissue [44]. Transcellular water transport is linked to a transport of potassium and chloride ions [47]. Water flow through the RMG and RPE cells membranes is facilitated by water-selective channels: the aquaporins. The major water channel of RPE cells and photoreceptors is aquaporin-1, whereas RMG cells express aquaporin-4 [48, 49]. Water transport is coupled to the spatial-buffering potassium currents flowing through RMG cells [50]. Alteration of the transglial water transport after downregulation of Kir4.1 channels and osmotic swelling of RMG cells under pathologic conditions such as transient retinal ischemia-reperfusion and diabetes mellitus have been implicated in the development of ME [51, 52].

Moreover, they contribute to the survival of ganglion cell neurons and photoreceptors, they are responsible for the stabilization of retinal structure, and they modulate inflammatory and immune responses [53, 54]. Thus, the RMG cells can upregulate the expression of inflammatory mediators, including MCP-1, which recruit microglial cells and phagocytotic monocytes/macrophages to regions of damage [55, 56]. Distinct disorders are associated with BRB breakdown, which results in the extravasation of the blood constituents that inactivate Kir channels and that induce RMG cell depolarization [57, 58].

Vascular leakage is a crucial pathogenic mechanism involved in ME [59]. Retinal capillaries are closely ensheathed by glial processes [53] and RMG cells enhance the barrier function of the vascular endothelium [60–62]. Due to inflammation and hypoxia, RMG cells produce factors such as VEGF, TNF-*α*, IL-1*β*, and prostaglandins, all of which enhance retinal vascular permeability [62–74].

Fluid clearance is usually mediated by osmotic water transport through RMG cells, a process facilitated by Kir channels and water channels, especially AQP4 [75–78]. AQP4 acts in combination with K^+^ channels to maintain osmotic retinal homeostasis. Indeed, Kir4.1 channel dysfunction, such as that observed in retinal vascular disorders, disturbs transcellular water transport [45, 79], resulting in water influx and RMG cells swelling [46]. Although a few studies have investigated the mechanisms of action of corticosteroids in ME, it has been shown that RMG cells express both the glucocorticoid receptor (GR) and the mineralocorticoid receptor (MR) [44]. Moreover, the MR ligand aldosterone increases the expression of AQP4 and Kir4.1, and it induces retinal swelling [80]. Finally, the two main corticosteroids used in intravitreal therapies, TA and dexamethasone, the latter administered through a sustained-release implant, regulate AQP4 and Kir4.1 distinctly, indicating that they are not functionally equivalent [44].

## 5. The Effect of the Different Intravitreal Therapies

It is also important to determine whether there are any differences in the response to different therapies. Intravitreal injection of both triamcinolone acetonide (TA) and bevacizumab has been reported to be effective in reducing macular thickness in DME [39, 81]. Indeed, intravitreal injection of TA is effective in decreasing macular thickness in patients with ME due to BRVO or CRVO, reducing the ocular expression of inflammatory cytokines [31]. Recently, it was shown that intravitreal TA injection significantly diminished MCP-1 (monocyte chemotactic protein-1) and MIP-1b (macrophage inflammatory protein-1b) levels in the aqueous humour of eyes with BRVO-ME [28]. Moreover, the decrease in aqueous humour MIP-1b, a chemokine with proinflammatory activity, was correlated with the basal foveal thickness and its improvement following TA injection. Although the exact mechanism leading to the improvement in BRVO-ME following intravitreal TA injection has not been well established, several possible mechanisms have been considered. For example, TA could downregulate VEGF, which might prevent a decrease in occlusion as well as inhibiting any increase in glial fibrillary acidic protein (GFAP) expression in RMG cells [82]. Likewise, intravitreal TA prevents osmotic swelling of the RMG cells through the opening of K^+^ (Kir) 4.1 channels and aquaporin-1 and aquaporin-4 (AQP-1 and -4) in the Müller cell membrane [83, 84]. These effects might reduce the BRB breakdown that occurs in BRVO, promoting the resolution of the ME. However, IL-6-independent VEGF secretion might also contribute to the persistence BRVO-ME after intravitreal TA injection [6].

Intravitreal injection of an anti-VEGF antibody has also been reported to be effective in reducing CRVO and DR associated with ME [39, 85]. Antiangiogenic drugs, such as ranibizumab, could be anti-inflammatory as well, and part of their actions could be through an anti-inflammatory process. They would need to be able to prevent the VEGF induced by TNF-*α* from acting on the RPE outside the cell. Inhibition of VEGF may act through both anti-inflammatory and antiangiogenic processes and human recombinant antiangiogenic isoforms such as VEGF-A_165_b can be anti-inflammatory on RPE cells stimulated by TNF-*α* [86].

While intravitreal TA injection may have the same beneficial effects as bevacizumab in decreasing foveal thickness and improving visual acuity in the management of ME due to BRVO, TA seems to be more effective than anti-VEGF therapy in patients with DME [23, 34]. Therefore, regarding the improvement in DME, anti-VEGF therapy would be less beneficial than corticosteroid therapy. This suggests that the pathogenesis of DME can be attributed not only to VEGF alone but also to the other inflammatory molecules that are suppressed by corticosteroids [31, 87]. Although the pathogenesis of DME is not fully understood, steroids can modulate vascular permeability by suppressing the expression of VEGF and its receptor, as well as IL-6 and ICAM-1. In addition, they can also reduce the activity of inflammatory cells that release cytokines, stabilizing cell membranes and tight junctions, acting upstream of pigment epithelium-derived factor (PEDF) expression [88]. Therefore, TA has multiple actions compared with bevacizumab, which only diminishes the intraocular levels of free VEGF.

The use of anti-VEGF and steroid agents in ME secondary to retinal vascular diseases is an evolving field. There is an ongoing debate regarding the safety, efficacy, and economic concerns related to these intravitreal therapies to reduce the treatment burden [89]. The future of treatment for DME and macular edema associated with central and branch retinal vein occlusion will probably be some kind of combination: anti-VEGF inhibitors, steroids, and laser.

In conclusion, inflammatory processes can be considered crucial in the pathogenesis of ME related to retinal vascular disorders, thereby representing important therapeutic targets in these diseases.
